# 956. Neurosurgical Infectious Disease Curriculum for Infectious Disease Fellows and Application of a Novel Surgical Infectious Disease Framework

**DOI:** 10.1093/ofid/ofab466.1151

**Published:** 2021-12-04

**Authors:** Jessica O’Neil, Christian Larsen, Molly L Paras

**Affiliations:** Massachusetts General Hospital, Boston, Massachusetts

## Abstract

**Background:**

Infectious disease (ID) consultations from surgical services account for 30-41% of all ID consults at academic medical centers. However, adult ID fellows in the United States complete residency training in Internal Medicine and may have limited prior exposure to patients on surgical services. We surveyed 16 first and second-year fellows of the combined Massachusetts General Hospital/Brigham and Women’s Hospital ID Fellowship to evaluate their self-perceived ability to approach ID consults from surgical services. While 75% self-reported confidence in their ability to approach general surgery consultations, only 33% reported confidence with neurosurgical related consultations.

**Methods:**

We created a novel framework for approaching surgical ID consult questions (Figure 1). We then developed two interactive case-based discussion sessions for first-year fellows to address common neurosurgical consult scenarios (post craniotomy/ craniectomy surgical site infections and cerebral spinal fluid shunt infections). The session materials, including images of common surgical approaches and risk factors for infection, were reviewed by a neurosurgeon content expert. An ID faculty member facilitated the discussions. Each discussion took place during a 30-minute teleconference. The learners then completed a self-assessment survey to evaluate the extent to which they could meet the educational objectives (Table 1) using a 1-5 Likert scale.

Figure 1. Surgical Infectious Diseases Framework

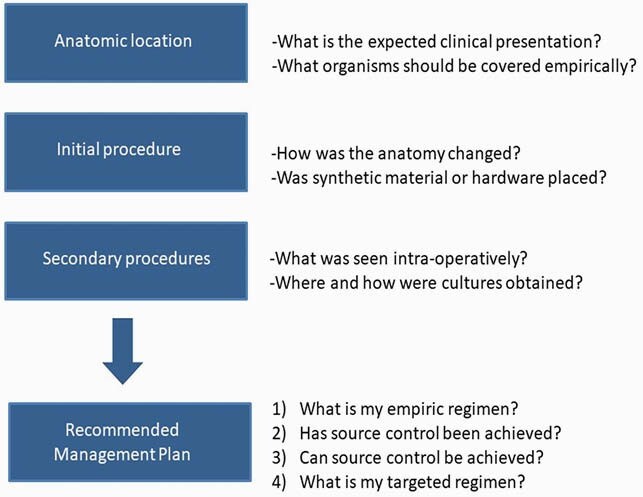

Table 1. Educational Objectives for Case 1 and 2

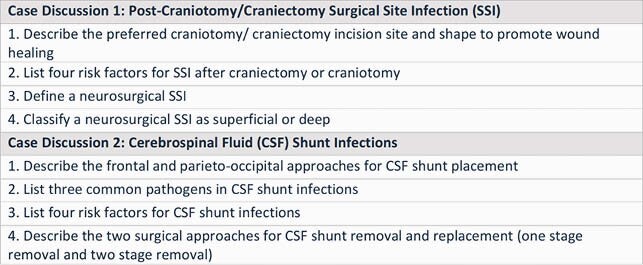

**Results:**

All sixteen learners (eight per case) completed the educational objective self-assessment surveys. The educational objectives were achieved with all questions reaching a mean response of 4 or greater indicating that the mean of learners agreed (4) or strongly agreed (5) that they were able to meet the outlined educational objectives after participating in the discussion session for Case 1 (Figure 2) and Case 2 (Figure 3).

Figure 2. Educational Objective Self-Assessment Scores for Case 1

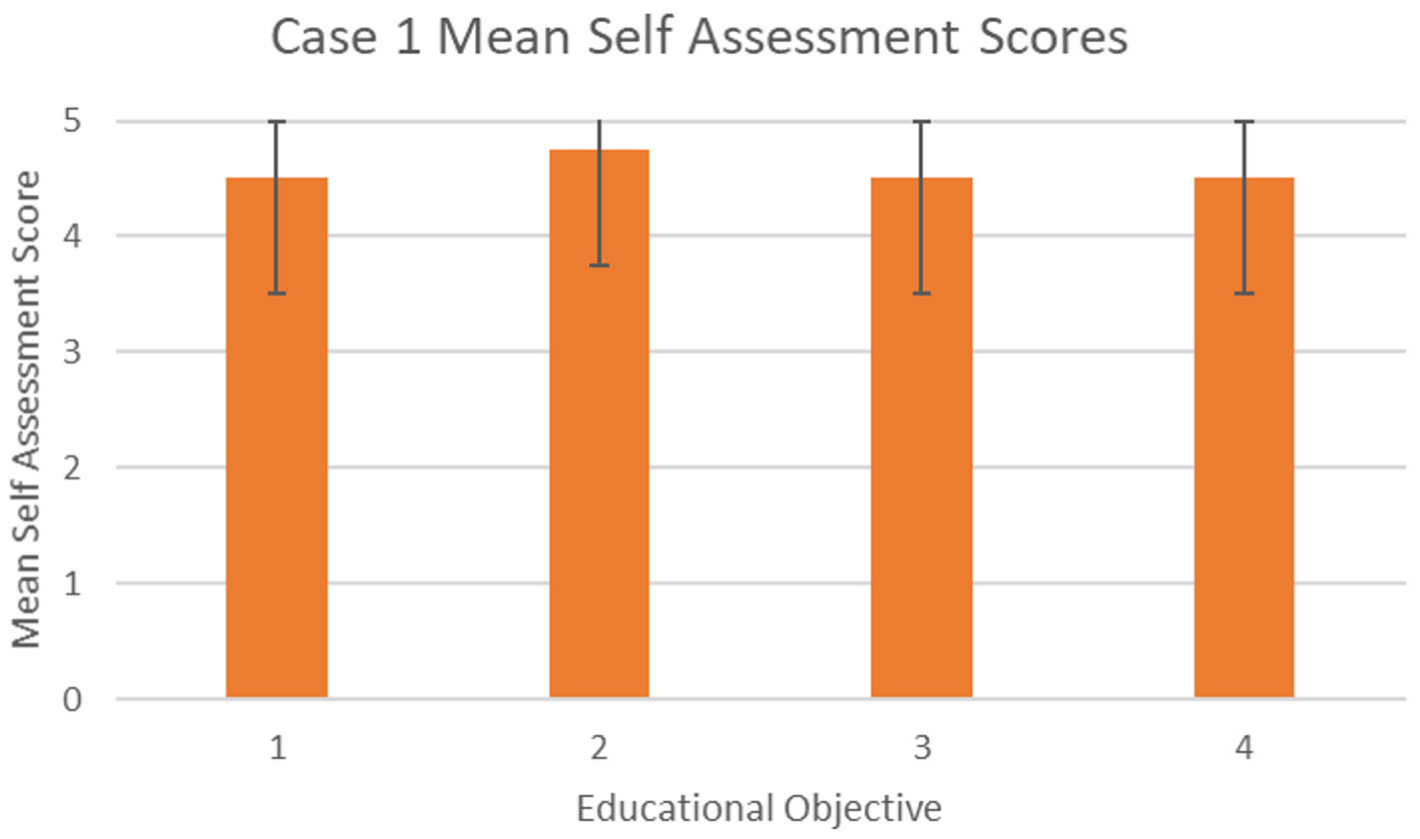

Figure 3. Educational Objective Self-Assessment Scores for Case 2

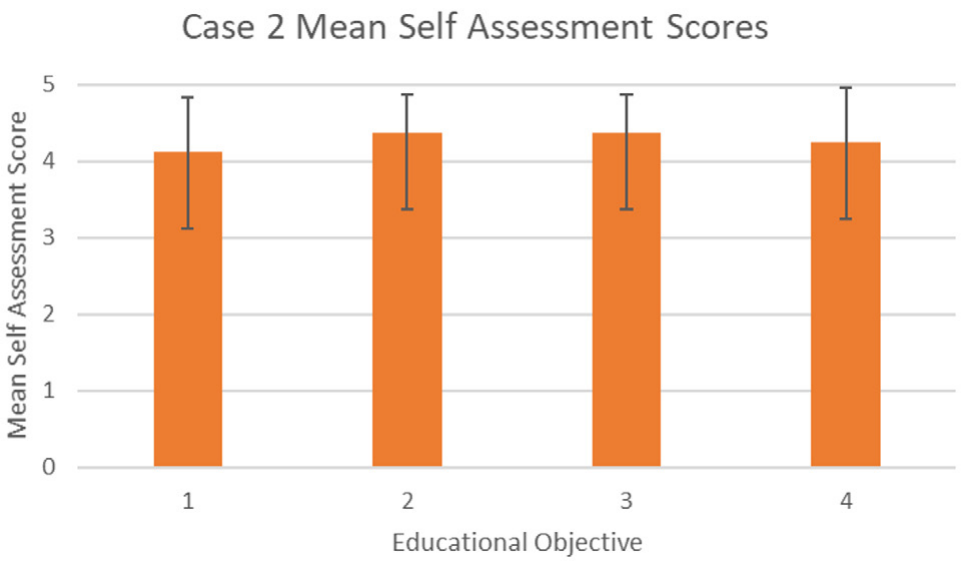

**Conclusion:**

Based on self-assessment surveys, our educational objectives were achieved. In turn, these first-year fellows may be better prepared to address ID consults from neurosurgical services in the future. While the case-based discussions were designed to address specific neurosurgical ID cases, our standardized framework could be adapted to a variety of surgical ID cases.

**Disclosures:**

**Molly L. Paras, MD**, **Deckermed** (Other Financial or Material Support, Payment for book chapter)

